# Hospitalisation, morbidity and outcomes associated with respiratory syncytial virus compared with influenza in adults of all ages

**DOI:** 10.1111/irv.12909

**Published:** 2021-12-01

**Authors:** Benjamin Andrew Leaver, Benjamin John Smith, Louis Irving, Douglas Forsyth Johnson, Steven Y. C. Tong

**Affiliations:** ^1^ Department of Respiratory Medicine Royal Melbourne Hospital Melbourne Victoria Australia; ^2^ Victorian Infectious Diseases Service Royal Melbourne Hospital Melbourne Victoria Australia; ^3^ Medicine, Dentistry and Health Sciences The University of Melbourne Melbourne Victoria Australia; ^4^ Department of General Medicine The Royal Melbourne Hospital Melbourne Victoria Australia

**Keywords:** adult, hospitalization, influenza human, morbidity, respiratory syncytial virus

## Abstract

**Background:**

Respiratory syncytial virus (RSV) is understood to be a cause of significant disease in older adults and children. Further analysis of RSV in younger adults may reveal further insight into its role as an important pathogen in all age groups.

**Methods:**

We identified, through laboratory data, adults who tested positive for either influenza or RSV between January 2017 and June 2019 at a single Australian hospital. We compared baseline demographics, testing patterns, hospitalisations and outcomes between these groups.

**Results:**

Of 1128 influenza and 193 RSV patients, the RSV cohort was older (mean age 54.7 vs. 64.9, *p* < 0.001) and was more comorbid as determined by the Charlson Comorbidity Index (2.4 vs. 3.2, *p* < 0.001). For influenza hospitalisations, the majority admitted were aged under 65 which was not the case for RSV (61.8% vs. 45.6%, *p* < 0.001). Testing occurred later in RSV hospitalisations as measured by the proportion tested in the emergency department (ED) (80.3% vs. 69.2%, *p* < 0.001), and this was strongly associated with differences in presenting phenotype (the presence of fever). RSV was the biggest predictor of 6‐month representation, with age and comorbidities predicting this less strongly.

**Conclusion:**

RSV is a significant contributor to morbidity and hospitalisation, sometimes outweighing that of influenza, and is not limited to elderly cohorts. Understanding key differences in the clinical syndrome and consequent testing paradigms may allow better detection and potentially treatment of RSV to reduce individual morbidity and health system burden. This growing area of research helps quantify the need for directed therapies for RSV.

## 1 INTRODUCTION

Respiratory syncytial virus (RSV) has increasingly been recognised for its capacity to cause significant morbidity in older adults. Research implicating the significance of RSV in elderly adults was first described in a series of outbreaks in nursing home facilities.^1^, ^2^ Subsequent ecological data demonstrated that during seasonal influenza and RSV peaks, there were increased hospitalisations and increased rates of morbidity amongst respiratory illness attributed hospitalisations; with this temporal link raising the possibility of a causal association between these occurrences.^3^, ^4^, ^5^ A case control study in 2018 demonstrated that patients older than 60 years of age hospitalised with RSV had a higher prevalence of comorbidities and worse morbidity and mortality outcomes when compared with influenza.^6^ The manifestations and implications of RSV in younger adult cohorts have not been characterised in depth.

It has been additionally hypothesised that the identification of RSV in individuals is often a by‐product of influenza testing; however, factors that contribute to this such as differences in clinical phenotype or testing paradigms have not been explored.^7^ Thus, there is a need to better understand the characteristics of RSV hospital presentations and admissions, including the clinical features, paradigms of testing and most importantly the mechanisms of RSV as a contributor to prolonged hospitalisation and re‐admission. Such an understanding will better delineate the place and role for targeted RSV therapeutics.

## 2 METHODS

### 2.1 Participants

Data were collected retrospectively from patients arriving via our emergency department who tested positive for either influenza A, B or RSV using combined polymerase chain reaction (PCR) testing on nasopharyngeal swab samples during a hospital presentation between January 2017 and June 2019 at a single centre university teaching hospital in Melbourne, Australia. Participants were included if they were 18 years of age or older, and all samples were tested in the same, single laboratory onsite at our centre with cases detected by a GeneXpert PCR panel. The introduction of GeneXpert, which provides a standardised panel of influenza A, B and RSV PCR results in all patients tested, was essential in allowing the detection of a standardised cohort free of selection bias. Previously, RSV and influenza PCR testing required separate requests and not all patients would be tested for both.

### 2.2 Data collection

A dataset was extracted using a combination of emergency department electronic medical records, laboratory test samples and administrative coding data and linked by matching unique testing identifiers with their corresponding unique patient hospital identifiers. The Charlson Comorbidity Index, a validated tool for quantifying multimorbidity, was calculated for each patient. Vital signs were extracted and recorded by the first documented set of observations on arrival to the emergency department, and additionally, the presence of fever in the emergency department was recorded.

Length of stay was collected and recorded in hours between point of presentation and discharge either directly from the emergency department or from an inpatient ward. Timing and location of diagnostic testing was recorded from laboratory coding data and linked to the unique corresponding patient and surrogate measures such as posttest length of stay was calculated from these metrics.

### 2.3 Data analysis

Data were collated and analysed using Stata version 13.0 for Windows statistical tools. Means and *T*‐tests were used for parametric data, rank‐sum tests for comparing medians of nonparametric data and chi^2^ was used to analyse categorical data. Correlation testing was performed using logistic regression for categorical‐dependent variables, and simple multivariate regression was used for continuous dependent variables. About 95% confidence intervals were used, and *p* values of less than 0.05 were considered significant.

## 3 RESULTS

**FIGURE 1:**
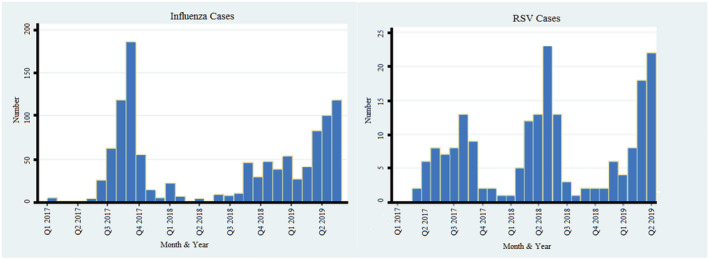
Longitudinal distribution of cases

A total of 1128 patients with influenza and 193 patients with RSV were identified (Figure 1).

**FIGURE 2:**
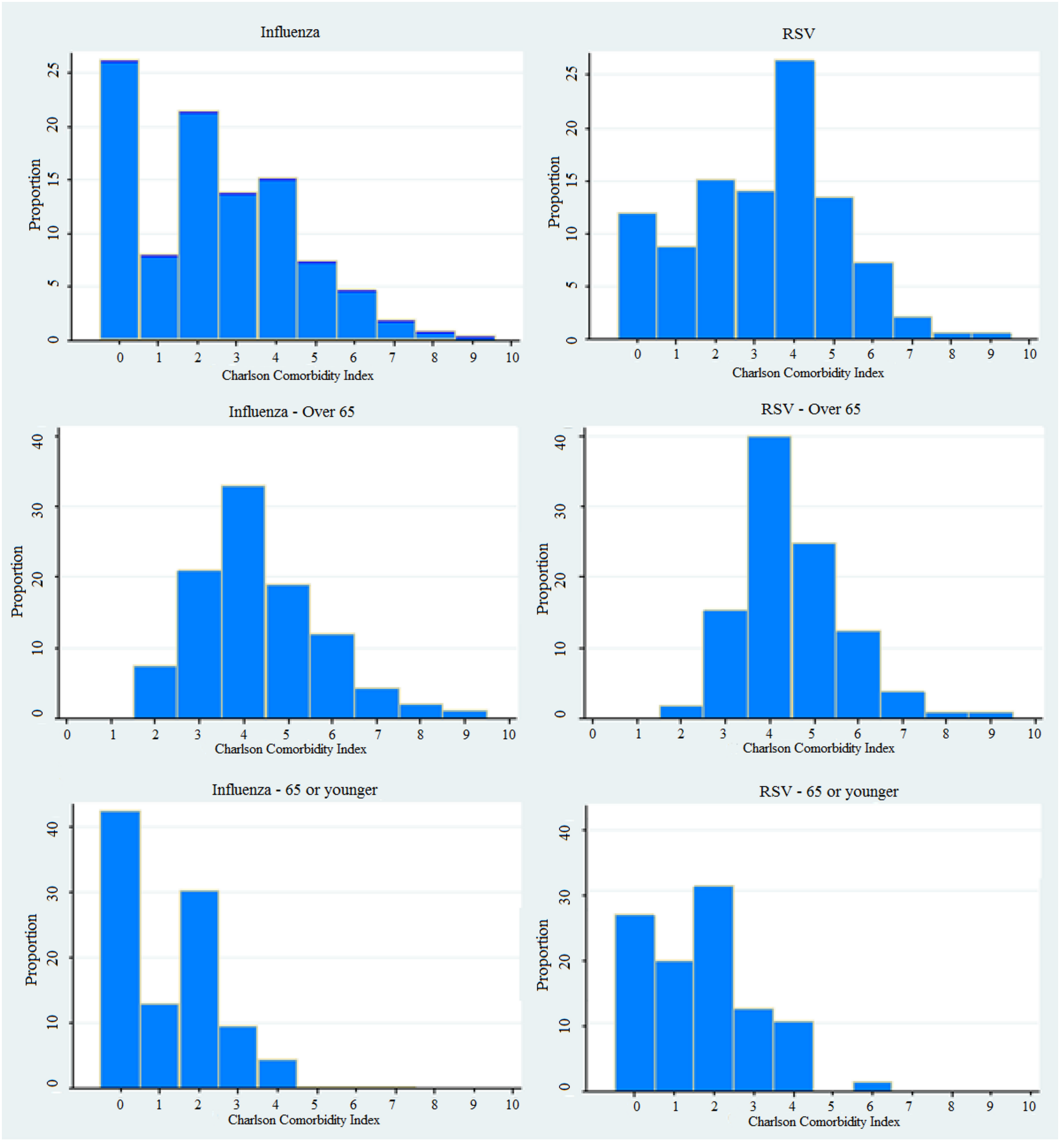
Charlson Comorbidity Index by age

The influenza cohort was significantly younger (mean age 54.7 vs. 64.9, *p* < 0.001) and had fewer comorbidities as measured by a lower average Charlson Comorbidity Index (mean 2.4 vs. 3.2, *p* < 0.001) (Figure 2). However, there was no significant difference seen in rates of hospital admission between the cohorts nor rates of ICU admission (see supporting information Tables S1 and S2).

**FIGURE 3:**
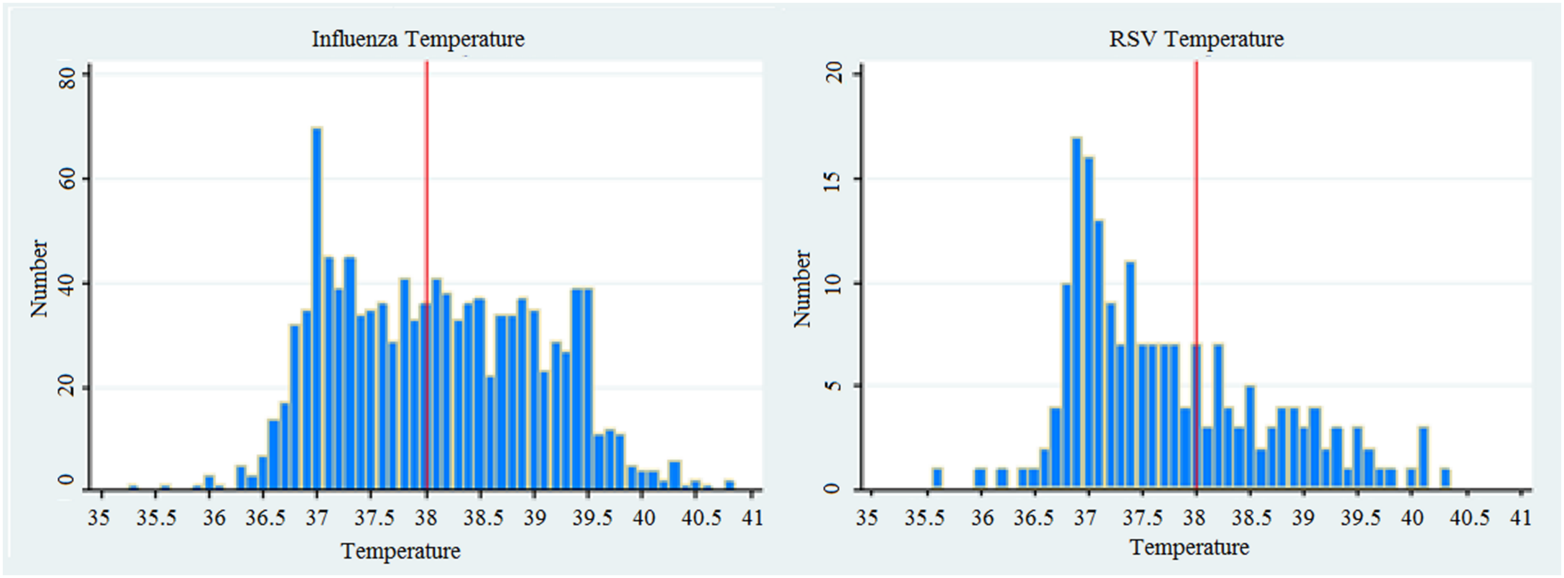
Comparative distribution of fever

Patients presenting with influenza were far more likely to be febrile at presentation (53.3% vs. 34.7%, *p* < 0.001) and hypotensive (10.9% vs. 5.7%, *p* = 0.027). A smaller proportion of patients with RSV presented febrile; instead, this cohort was more likely to be tachypnoeic (47% vs. 56%, *p* = 0.021). Rates of tachycardia and hypoxia were similar between groups (see Table 1 and Figure 3).

**TABLE 1:** Abnormal observations

	Influenza	RSV	*p* value
Fever (% of total ≥38°C)	601 (53.3%)	67 (34.7%)	<0.001
Tachycardia (% of total ≥100 BPM)	474 (42.0%)	80 (41.5%)	0.882
Hypoxia (% of total saturations ≤92%)	50 (4.4%)	8 (4.2%)	0.857
Tachypnoea (% of total respiratory rate ≥20)	530 (47.0%)	108 (56.0%)	0.021
Hypotensive (% of total SBP ≤ 100)	123 (10.9%)	11 (5.7%)	0.027

Fever in over 65 (% of total ≥38°C)	184 (42.7%)	33 (31.4%)	0.035
Fever in 65 or under (% of total ≥38°C)	417 (59.8%)	34 (38.6%)	<0.001

Tachypnoea in over 65	253 (58.7%)	66 (62.9%)	0.437
Tachypnoea in 65 or under	277 (39.7%)	42 (47.7%)	0.151

Abbreviation: RSV, respiratory syncytial virus.

Those under 65 presenting with influenza were significantly more likely to be febrile than those over 65 (59.8% vs. 42.7%, *p* < 0.001), whereas there was no significant difference in rates of fever across age groups presenting with RSV (Figure 3). In both influenza and RSV presentations, those over the age of 65 were more likely to be tachypnoeic than those under 65.

Influenza patients were more likely to be tested in the emergency department, whereas RSV patients were more likely to be tested later in the admission (80.3% vs. 69.2%, *p* < 0.001) (see Table 2).

**TABLE 2:** Testing patterns

	Influenza	RSV	*p* value
Proportion tested in ED	906 (80.3%)	137 (69.2%)	<0.001

	Febrile	Afebrile
Tested in ED	565 (84.6%)	478 (73.2%)
Tested beyond ED	103 (15.4%)	175 (26.8%)

	Tachypnoeic	Normal Respiratory Rate	*p* value
Tested in ED	513 (80.4%)	530 (77.6%)	0.211
Tested beyond ED	125 (19.6%)	153 (22.4%)	0.211

The length of stay for the influenza cohort was significantly shorter than that of RSV with a similar difference in posttest length of stay. It follows that the proportion of stays longer than 1 week was also found to be higher in the RSV subgroup (Table 3).

**TABLE 3:** Length of stay

	Influenza	RSV	*p* value
Length of stay (h)	61.4 (55.0–67.7)	100.3 (78.3–122.4)	<0.001
Post‐test length of stay (h)	54.0 (48.1–59.8)	91.9 (70.4–113.3)	<0.001
Length of stay > 1 week (% of total)	91 (8.1%)	29 (15.0%)	0.002

Abbreviation: RSV, respiratory syncytial virus.

**FIGURE 4:**
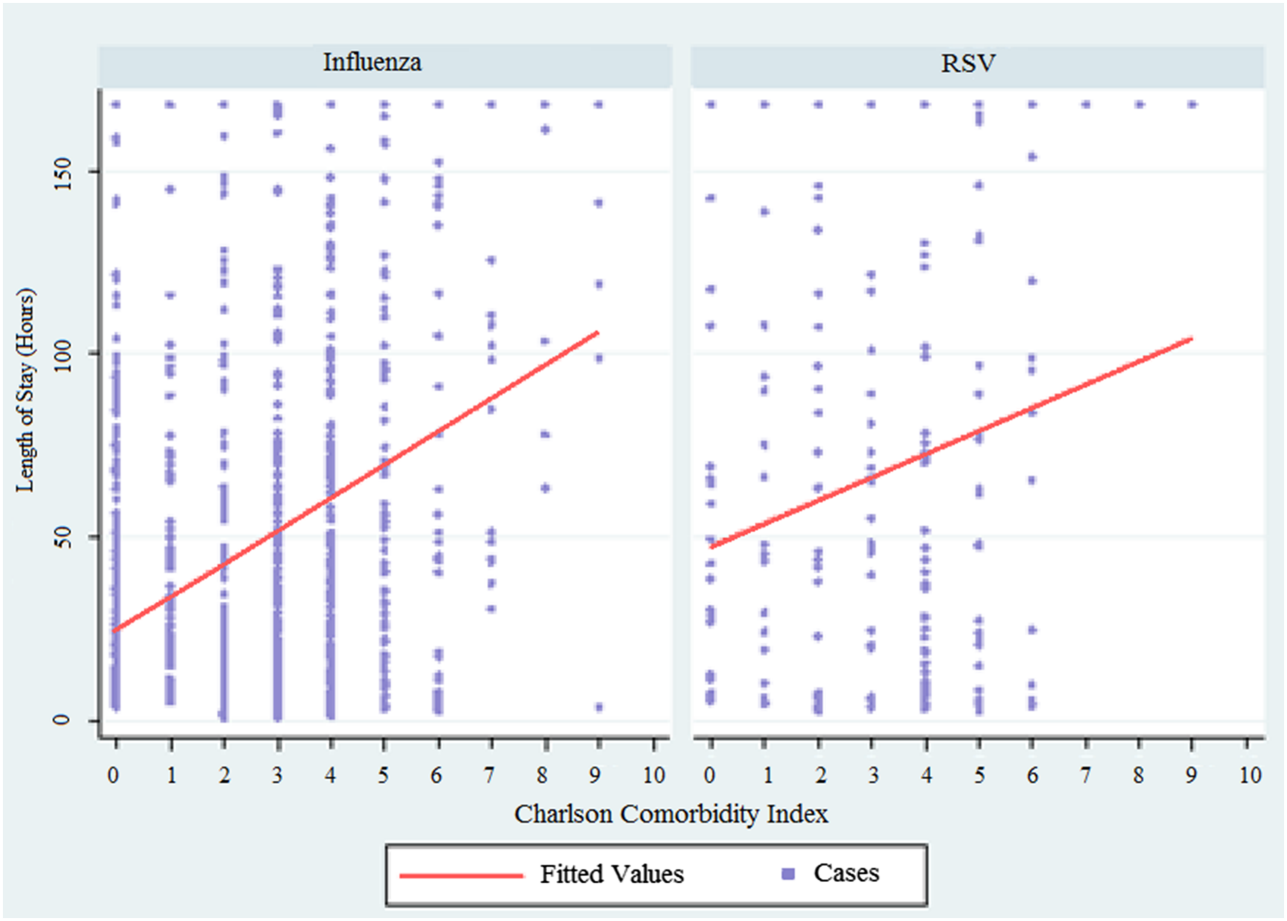
Charlson Comorbidity Index versus length of stay

**FIGURE 5:**
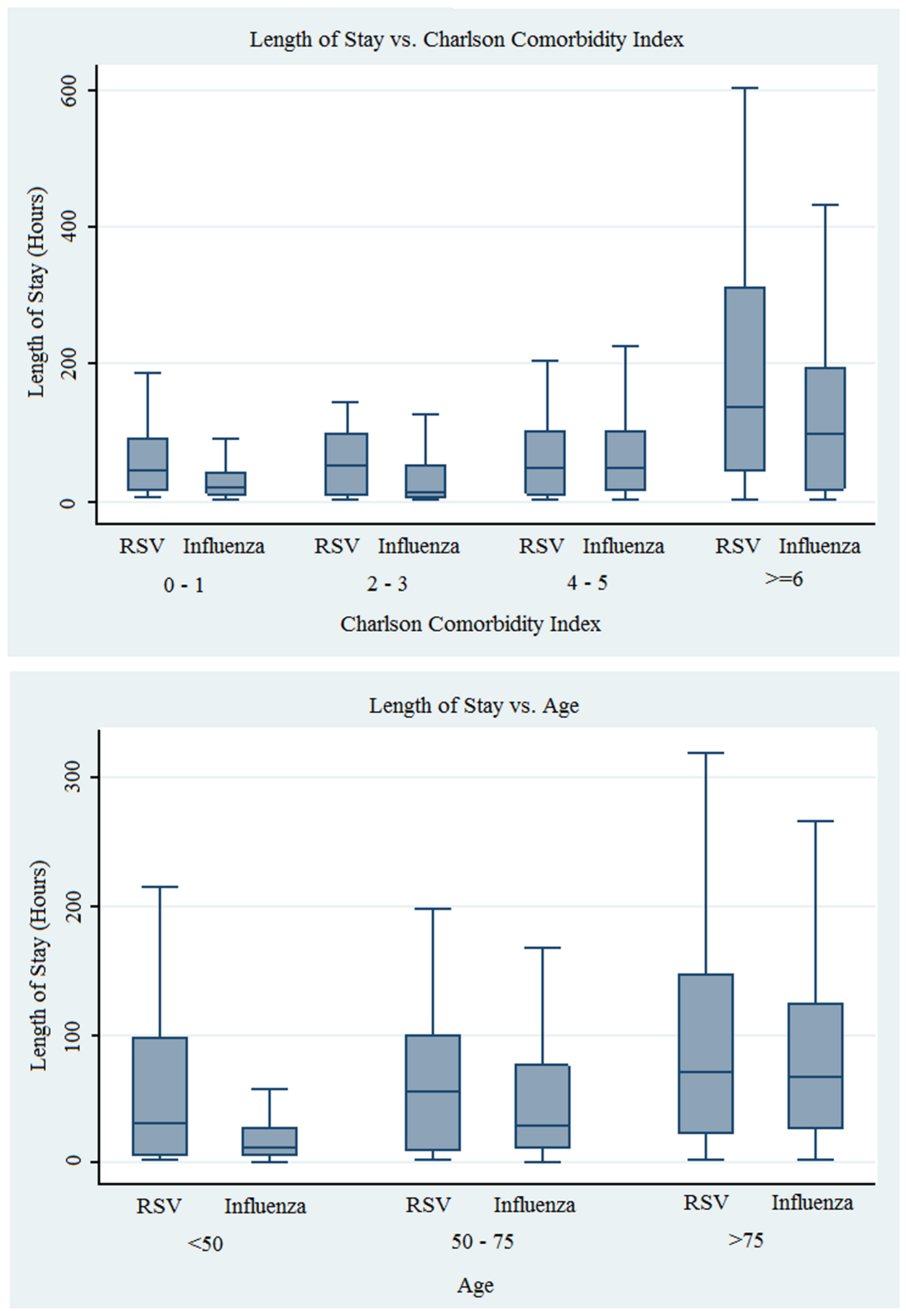
Length of stay versus grouped Charlson Comorbidity Index and age

For both cohorts, admission to ICU and the Charlson Comorbidity Index was found to have a strong effect on length of stay. For patients with influenza, age, gender and additionally heart rate were also found to an impact. Other vital signs, including temperature and respiratory rate, did not have an associated impact. In those with RSV, there was no statistical association between age, gender, vital signs and an impact on length of stay (see supporting information Table S2, Table 3, Figure 4 and Figure 5).

Having influenza and being admitted to ICU in first admission were strong predictors of a lower rate of 6‐month representation as measured by odds ratio. Our analysis additionally revealed that the initial length of stay and age both had an effect on 6‐month re‐admission though their quantifiable effect that was negligible. Rate of comorbidities was not a predictor of either 30‐day or 6‐month readmission, and further, our model did not reveal predictors of 30‐day readmission (see supporting information Table S3).

Both groups had similar rates of mortality and discharge to a location other than home (either a subacute hospital for ongoing care or a nursing home) (see Table 4).

**TABLE 4:** Hospital disposition

	Influenza	RSV	*p* value
30‐day representation	93 (8.3%)	16 (8.3%)	0.983
6‐month representation	169 (15.0%)	52 (27.0%)	<0.001
Disposition not home, not death	66 (5.9%)	15 (7.8%)	0.304
Death	14 (1.2%)	2 (1.0%)	0.810

Abbreviation: RSV, respiratory syncytial virus.

## 4 DISCUSSION

This Australian retrospective cohort examined the period of January 2017 through June 2019 and explored the role of RSV as a significant contributor, when compared with influenza, to hospitalisations, prolonged bedstay, morbidity and mortality outcomes. The study also explores differences in key presenting characteristics and draws a link to differences in testing practices. Finally, the paper demonstrates key insight into the burden and characteristics of RSV disease compared with influenza in adults under the age of 65, whereas the existing literature has largely focused on cohorts over 65.

The longitudinal distribution of both influenza and RSV cases demonstrated a seasonal peak in both viruses with the majority of cases in both instances being identified in the winter periods. The exception to this was seen in the 2018 data where proportionally very few influenza cases were identified, consistent with low cases reported nationally during this period.^8^ Based on larger ecological data sets modelled in the United States, it has been hypothesised that a significant proportion of RSV was detected as a by‐product of influenza directed testing and consequently suggested that RSV may not follow seasonal influenza trends so concordantly and may instead be under detected during hotter months when influenza has lower incidence.^7^ Curiously, our data demonstrating peak RSV caseloads during the 2018 quiet influenza season suggest very strongly that testing patterns, triggers for testing and clinical detection of RSV are far more complex, and RSV may in fact follow similar seasonal trends to influenza with the peak occurring slightly earlier.

Concordant with existing research, the mean age of our RSV cohort was significantly higher than that of influenza.^9^ The majority of the hospital presentations with influenza were under the age of 65, compared with RSV where the over 65 group had a slight majority. It follows that the proportional majority of influenza admissions were aged under 65 when compared with RSV (majority were over 65). Hypothesising that there are many variables contributing to this, our research did demonstrate that presentation with influenza was less discriminatory on age and comorbidity when compared with RSV. Further, selection bias may also contribute that would be better explored with community versus hospitalised infection data. However, this does not negate an important observation that in an individual under 65 presenting to hospital with either virus, admission to hospital or ICU occurred at similar frequency. This highlights the notion that RSV represents a significant pathogen as a cause of hospitalisation. Higher 6‐month representation rates and similar rates of death and other morbidity indices when compared with influenza further this concept. There was a significantly higher prevalence of key comorbidities in the RSV group, notably diabetes mellitus, chronic obstructive pulmonary disease and congestive heart failure. This was also reflected in the Charlson Comorbidity Index as an overall measure of comorbidity burden which was again seen to be statistically higher in the RSV subgroup.

Lengths of stay as measured in hours, as well as prolonged hospitalisation as measured by proportion of those hospitalised for greater than 1 week, were markedly higher in the RSV cohort. Regression analysis revealed that for the RSV subgroup, the overall burden of comorbidities was the strongest predictor of length of stay, as was being admitted to the ICU rather than a ward bed. However, other factors such as age did not appear to predict length of stay in the RSV cohort, with two possible hypotheses for this finding. The first possibility is that our RSV group was too small to detect a real impact of age on length of stay, which is one limitation of this data. Our much larger influenza cohort did in fact detect age as a statistically significant determinant of length of stay; however, the regression coefficient as a measure of quantitative impact of age in this influenza group was far less impressive than might be expected. This raises an alternative hypothesis that younger patients presenting to our hospital with RSV suffer an equally significant burden of illness when compared with their more elderly RSV counterparts, adding to the evidence that RSV is an important pathogen leading to hospitalisation and morbidity not necessarily isolated to the very elderly and more comorbid populations.

One limitation of this paper is that it did not explore the impact of targeted therapeutics on length of stay. One hypothesis may be that as RSV lacks readily available targeted therapies when compared with influenza where, in Australia, oseltamivir is commonly prescribed to hospitalised adults that this may contribute to the longer length of stay seen in RSV admissions. This association could be explored in future research and would add significantly to the argument for the need for directed therapies against RSV.

One unexpected finding of our regression analysis was the impact of gender on length of stay in the influenza subgroup. There was a strong correlation between being male and a longer length of stay, though with wide confidence intervals for this association. No such finding was seen in the RSV population. This is despite relatively similar proportions of males and females in both groups.

An important pattern demonstrated in this paper was the key comparative differences in clinical phenotype between influenza and RSV. To our knowledge, there is only very limited data exploring this comparison,^9^ and our paper is one of the first to link key presenting phenotypes with diagnostic patterns and timing of PCR testing.^10^ We found that those presenting with influenza were far more likely to be febrile, whereas only a minority of RSV patients were febrile. The RSV group was significantly more likely to be tachypnoeic and less likely to be shocked as indicated by systolic hypotension. Influenza was much more likely to be detected in the emergency department, whereas a greater proportion of RSV patients had their infection detected later in the admission beyond the emergency department. We demonstrated that fever was a strong predictor of being tested in the emergency department, whereas tachypnoeic did not predict earlier testing. This pattern does support the earlier hypothesis that RSV in many instances may only be detected as a by‐product of influenza testing, and the true incidence in hospitalised patients may be proportionally underestimated. It also highlights a salient point that other identifiers of possible respiratory viral illness, such as tachypnoea, should be used to judge whether a patient may be affected by a virus such as RSV. The impact of changes in patterns of testing to better control this variable may lead to significant implications both directly and indirectly on earlier RSV disposition from emergency department (ED) or hospital, overall length of stay and perhaps even reduce detected or undetected nosocomial transmission to patients and staff.

Representation rates did not differ at 30 days; however, significant discrepancy appeared at 6 months with more than a quarter of RSV patients representing. Logistic regression revealed, as expected, that RSV was the strongest predictor of 6‐month representation. Age, initial admission requiring ICU and the initial length of stay were all also predictors but only minimally contributed. Overall comorbidity burden did not predict either point of representation which was an unexpected finding. This raises the hypothesis that presenting to hospital with RSV is an important historical point to recognise in a patient's history as the manner in which it contributes to this representation rate appears far more complex than simply being attributable to older age or being a more comorbid patient. Whether there are compounding variables at play or not, this could be further explored in future research looking into comparative differences of community versus hospitalised RSV infection. This may also lead to a better understanding of indications for using RSV directed therapeutics.

## 5 CONCLUSION

We have found that RSV is an important contributor to prolonged hospitalisation and representation in adults when compared with influenza. Further, this paper shows that the burden and impacts of this illness are not simply linked to being more elderly or more comorbid. The data also support that in some, but not all, instances, RSV may be only detected as a by‐product of testing for influenza, and given that it has a significantly different clinical phenotype, it not only may be underdetected but that it is often detected later in hospitalisations. This has implications for diagnostic and therapeutic pathways, infection control and system‐level health burden. This paper entices the need for further research into RSV in adults, particularly into characterising the mechanisms that cause the key discrepancies when compared with influenza. Finally, our paper adds to a growing body of evidence that RSV can cause important illness in adults of all ages and highlights potential benefits for both individuals and health systems that the development of targeted therapeutics may bring.

## AUTHOR CONTRIBUTIONS

**Benjamin John Smith:** Conceptualization; data curation; formal analysis; software; supervision. **Louis Irving:** Conceptualization; methodology; supervision. **Douglas Forsyth Johnson:** Conceptualization; investigation; methodology; supervision. **Steven Tong:** Conceptualization; investigation; methodology; supervision.

## CONFLICT OF INTEREST

There are no conflicts of interest.

### PEER REVIEW

The peer review history for this article is available at https://publons.com/publon/10.1111/irv.12909.

## Supporting information


**TABLE S1** Key baseline demographics and comorbidities
**TABLE S2** Multivariate regression of length of stay (hours)
**TABLE S3** Representation rates

## Data Availability

The data that support the finding of this study are available in the supporting information of this article.
