# Supplementary material for: Hospitalisation, morbidity and outcomes associated with respiratory syncytial virus compared with influenza in adults of all ages
Source: Influenza Other Respir Viruses. 2021 Dec 1;16(3):474–80. doi: 10.1111/irv.12909 (PMC8983890; doi:10.1111/irv.12909)
Supplement: Supplementary file 1 — TABLE S1 Key baseline demographics and comorbidities TABLE S2 Multivariate regression of length of stay (hours) TABLE S3 Representation rates [file IRV-16-474-s001.docx]

| *SUPPLEMENTARY* *TABLE 1 - KEY BASELINE DEMOGRAPHICS AND COMORBIDITIES* | | | |
| --- | --- | --- | --- |
|  | Influenza | RSV | p value |
| Total Number | 1128 | 193 |  |
| Age | 54.7 (53.4 – 56.1) | 64.9 (62.2 – 67.6) | <0.001 |
| Male | 596 (52.8%) | 83 (43.0%) | 0.285 |
| Comorbidities |  |  |  |
| Diabetes Mellitus | 155 (13.7%) | 37 (19.2%) | 0.048 |
| Chronic Obstructive Pulmonary Disease | 114 (10.1%) | 38 (19.7%) | <0.001 |
| Chronic Kidney Disease | 55 (4.9%) | 15 (7.8%) | 0.070 |
| Ischaemic Heart Disease | 15 (1.3%) | 3 (1.6%) | 0.804 |
| Congestive Heart Failure | 48 (4.3%) | 19 (9.9%) | 0.001 |
| Chronic Liver Disease | 3 (0.3%) | 1 (0.5%) | 0.556 |
| Human Immunodeficiency Virus | 1 (0.1%) | 0 (0%) | 0.679 |
| Malignancy | 11 (1.0%) | 2 (1.0%) | 0.937 |
| Charlson Comorbidity Index | 2.4 (2.3 – 2.5) | 3.2 (2.9 – 3.5) | <0.001 |
| Admission Characteristics |  |  |  |
| Proportion Admitted | 852 (75.5%) | 158 (81.9%) | 0.055 |
| Proportion Admitted ICU | 33 (2.9%) | 9 (4.7%) | 0.204 |
| Admissions - OVER 65 |  |  |  |
| Total Number (% of total virus) | 431 (38.2%) | 105 (53.4%) | <0.001 |
| Admitted (% of total over 65) | 362 (84.0%) | 90 (85.7%) | 0.663 |
| Admitted ICU (% of total over 65) | 14 (3.3%) | 4 (3.8%) | 0.775 |
| Admissions - 65 OR YOUNGER |  |  |  |
| Total Number (% of total virus) | 697 (61.8%) | 88 (45.6%) | <0.001 |
| Admitted (% of total 65 or younger) | 490 (70.3%) | 68 (77.3%) | 0.174 |
| Admitted ICU (% of total 65 or younger) | 19 (2.7%) | 5 (5.7%) | 0.129 |

| *SUPPLEMENTARY TABLE 2 – MULTIVARIATE REGRESSION OF LENGTH OF STAY (HOURS)* | | | | | | |
| --- | --- | --- | --- | --- | --- | --- |
| *MULTIVARIATE REGRESSION - INFLUENZA* | | | | | | |
|  | Coefficient | Std. Err. | T | p value | 95% confidence interval | |
| Age | 1.077 | 0.394 | 2.74 | **0.006** | 0.305 | 1.850 |
| Gender: male | 12.612 | 6.11 | 2.06 | **0.039** | 0.623 | 24.602 |
| Admitted to ICU | 206.435 | 17.880 | 11.55 | **<0.001** | 171.352 | 241.517 |
| Heart Rate | 0.590 | 0.241 | 2.45 | **0.014** | 0.118 | 1.062 |
| Systolic Blood Pressure | 0.084 | 0.181 | 0.47 | 0.641 | -0.270 | 0.438 |
| Respiratory Rate | 1.669 | 0.908 | 1.84 | 0.066 | -0.112 | 3.449 |
| Temperature | -1.750 | 6.009 | -0.29 | 0.771 | -13.539 | 24.887 |
| Oxygen Saturation | -0.464 | 1.456 | -0.32 | 0.750 | -3.320 | 2.392 |
| Charlson Comorbidity Index | 5.380 | 2.618 | 2.05 | **0.040** | 0.242 | 10.517 |
|  | R-squared = 0.200 Adjusted R-squared = 0.1939  Prob(F-statistic) = 0.000 | | | | | |
| *MULTIVARIATE REGRESSION – RSV* | | | | | | |
|  | Coefficient | Std. Err. | T | p value | 95% confidence interval | |
| Age | 0.851 | 1.682 | 0.51 | 0.613 | -2.467 | 4.170 |
| Gender: male | -5.481 | 22.849 | -0.24 | 0.811 | -50.574 | 39.612 |
| Admitted to ICU | 261.567 | 55.826 | 4.69 | **<0.001** | 151.421 | 371.712 |
| Heart Rate | -1.462 | 1.032 | -1.42 | 0.158 | -3.500 | 0.575 |
| Systolic Blood Pressure | -0.024 | 0.566 | -0.04 | 0.966 | -1.141 | 1.092 |
| Respiratory Rate | -2.370 | 3.048 | -0.78 | 0.438 | -8.386 | 3.645 |
| Temperature | 17.451 | 23.832 | 0.73 | 0.465 | -29.580 | 64.484 |
| Oxygen Saturation | -6.691 | 3.525 | -1.90 | 0.059 | -13.647 | 0.264 |
| Charlson Comorbidity Index | 25.066 | 10.083 | 2.49 | **0.014** | 5.167 | 44.965 |
|  | R-squared = 0.0662 Adjusted R-squared = 0.0256  Prob (F-statistic) = 0.1186 | | | | | |

| *SUPPLEMENTARY TABLE 3 – REPRESENTATION RATES* | | | | | | |
| --- | --- | --- | --- | --- | --- | --- |
| 30 DAY REPRESENTATION RATE LOGIRTICAL REGRESSION | | | | | | |
|  | Odds Ratio | Std. Err. | Z | p value | 95% confidence interval | |
| Virus: influenza | 1.103 | 0.317 | 0.34 | 0.733 | 0.628 | 1.936 |
| Age | 1.014 | 0.008 | 1.62 | 0.104 | 0.997 | 1.030 |
| Gender: male | 1.284 | 0.259 | 1.24 | 0.217 | 0.864 | 1.907 |
| Admitted to ICU | 1.461 | 0.749 | 0.74 | 0.460 | 0.535 | 3.989 |
| Initial length of Stay | 1.001 | 0.002 | 0.27 | 0.785 | 0.997 | 1.005 |
| Charlson Comorbidity Index | 0.963 | 0.081 | -0.44 | 0.662 | 0.817 | 1.137 |
| 6 MONTH REPRESENTATION RATE LOGITICAL REGRESSION | | | | | | |
| Virus: influenza | 0.585 | 0.111 | -2.83 | **0.005** | 0.404 | 0.848 |
| Age | 1.015 | 0.006 | 2.40 | **0.016** | 1.003 | 1.028 |
| Gender: male | 1.050 | 0.161 | 0.32 | 0.748 | 0.778 | 1.418 |
| Admitted to ICU | 0.299 | 0.164 | -2.20 | **0.027** | 0.102 | 0.874 |
| Initial length of Stay | 1.006 | 0.001 | 4.28 | **<0.001** | 1.003 | 1.009 |
| Charlson Comorbidity Index | 0.976 | 0.061 | -0.38 | 0.702 | 0.863 | 1.105 |
